# Distinct roles of anterior cingulate and subcortical regions supporting effort evaluation versus preparation

**DOI:** 10.1162/IMAG.a.1417

**Published:** 2026-10-30

**Authors:** Nanne Kukkonen, Senne Braem, Joshua O. Eayrs, Mengqiao Chai, Stefania Mattioni, S. Tabitha Steendam, C. Nico Boehler, Jan R. Wiersema, Wim Notebaert, Ruth M. Krebs

**Affiliations:** Department of Experimental Psychology, Ghent University, Ghent, Belgium; Research Centre for Brain and Behaviour, School of Psychology, Liverpool John Moores University, Liverpool, United Kingdom; Department of Experimental Clinical and Health Psychology, Ghent University, Ghent, Belgium

**Keywords:** cognitive effort, effort evaluation, effort preparation, ACC, midbrain, pupillometry

## Abstract

Cognitive effort encompasses the mental exertion invested to achieve a goal as well as the experienced cost associated with that exertion. The dopaminergic midbrain regions (i.e., ventral tegmental area/substantia nigra), ventral striatum, and anterior cingulate cortex have all been broadly implicated in effort regulation. However, less is known about the role of these regions in dissociable subprocesses such as effort evaluation versus effort preparation. In this preregistered functional MRI study with concurrent pupil tracking, we disentangled the evaluation and preparation phase of reward- and effort-based motivation. In the evaluation phase, the typical valuation regions encoded high-reward and low-effort demand as motivational factors. In the preparation phase, the anterior cingulate cortex displayed increased activity for demanding high-reward trials. The dopaminergic midbrain exhibited a positive reward effect only during task execution. The results suggest that when separating evaluation from preparation, regions traditionally thought of as primarily reward attuned respond to high reward and low effort during evaluation but can assign a motivating signal to effort during preparation, while the anterior cingulate cortex integrates effort and reward prospect to subserve only preparatory control processes.

## 1 Introduction

Cognitive effort involves the allocation of mental resources toward a goal, allowing us to navigate through life’s complexities with effective problem solving and learning. Effort varies across individuals (Treadway et al., 2012) and difficulties with cognitive effort regulation are symptomatic of a variety of conditions such as chronic fatigue syndrome (Walitt et al., 2024) and ADHD (Sergeant, 2005; Wagner et al., 2024). Effort is commonly experienced as aversive (David et al., 2022), preferentially avoided (Kool et al., 2010), and typically conceptualized as a cost in neuroeconomic models of decision making (Kool & Botvinick, 2018; Shenhav et al., 2013; Verguts et al., 2015). However, it is sometimes also preferred over idleness (Wu et al., 2023) and can be considered as something positive once it has been expended (Clay et al., 2022; Inzlicht et al., 2018). Similarly, studies investigating neural dynamics of effort-related processes have reported effort as a reward-discounting cost in the brain during effort-based decision making (Lopez-Gamundi et al., 2021; Westbrook et al., 2019), as well as motivating effects of effort expectations during effort preparation (Krebs et al., 2012; Vassena et al., 2014). In this study, we systematically studied how different effort-related regions are implicated in both effort evaluation versus preparation within the same paradigm.

During value-based decision making, incentive values are thought to be integrated with a variety of costs in a domain-general valuation system, comprising cortical and subcortical regions, with primary loci in the ventral striatum (VS), and the ventromedial prefrontal cortex (vmPFC; Bartra et al., 2013). Activity in these regions has been shown to scale with incentive value discounted by both the risk and the delay of payoff (Peters & Büchel, 2009), and by monetary losses (Basten et al., 2010). Specifically, various costs correlate negatively with activity in these regions, resulting in a net value representation of an option, that is, the outcome value discounted by the cost associated with procuring it. While these regions have also been implicated in representing the net value of physical effort during decision making (Aridan et al., 2019; Arulpragasam et al., 2018; Hogan et al., 2019), fMRI investigations of cognitive effort valuation have yielded inconsistent results. Some studies implicated the anterior cingulate cortex (ACC) in representing the net value of effortful rewards (Chong et al., 2017; Massar et al., 2015), while others found ACC activity to reflect effort choice difficulty rather than effort value (Westbrook et al., 2019).

Westbrook et al. (2019) also found increasing subjective value of effort to be associated with increasing activity in VS and vmPFC. These results were corroborated by a meta-analysis of fMRI studies investigating valuation of both cognitive and physical effort, involving paradigms with and without a choice component (Lopez-Gamundi et al., 2021). The authors found that both vmPFC and, less conclusively, VS track the net value of effort, while ACC was positively correlated with the effort demand level. The less consistent role of VS in effort-based outcome valuation might be due to the functionally heterogenous subregions of VS assigning opposing signs to effort (Suzuki et al., 2021). Based on these findings, the cost–benefit calculations involving effort seem to be partly subserved by the same neural structures that map costs from different sources to a common scale (Levy & Glimcher, 2012), hypothetically enabling direct comparison between effort costs and costs from other sources.

ACC is involved in a multitude of cognitive control processes, subserving performance monitoring and adjustments across a range of tasks (Nee et al., 2011; Niendam et al., 2012; Ridderinkhof et al., 2004; Shackman et al., 2011). The expected value of control model (EVC) posits that ACC (ACC; specifically dorsal ACC) tracks effort–value calculations and detects the need for control, dynamically orchestrating adaptations in control signals based on this need (Shenhav et al., 2013; 2016). Extending the EVC account, in the Reinforcement Meta-Learner model (RML; Silvetti et al., 2018), ACC (dorsal) comprises two functionally distinct modules: an Act module for action selection toward the external environment and a Boost module that learns the optimal level of catecholamine release from the brainstem nuclei (ventral tegmental area and locus coeruleus) for a given environmental state. The Boost module positions ACC as an active meta-learner that allows for behavior optimization under varying demands, accounting for the plasticity of effort-related decision making.

In addition to responding to the costs and benefits of expected effort, ACC activity is also linked to retroactive effort valuation: in a study by Botvinick et al. (2009), VS response to reward feedback was negatively correlated with activity in ACC during task engagement, suggesting that ACC codes the inherent cost of effort that, in turn, devalues its outcomes in value-responsive regions. Indeed, within the EVC model, ACC is a hub of effort-based decision making and effort allocation that is functionally distinct from other regions that subserve stimulus valuation or execution of control. Lesion studies in monkeys (Rudebeck et al., 2008) and humans (Camille et al., 2011) similarly suggested a division of labor between ACC and valuation areas by uncovering a double-dissociation between the orbitofrontal cortex (OFC; overlapping structurally and functionally with vmPFC) and ACC. OFC disruptions were associated with impaired stimulus-value learning, leaving action-value learning intact, while ACC lesions led to a selective action-value learning deficit.

To complement effort-based decision making and computational accounts, experimental studies have probed the neural underpinnings of anticipatory effort in absence of an explicit decision of whether to exert effort. In these studies, when both expected reward and expected effort were signaled by cues prior to task engagement, effort prospect has been found to have a motivating effect similar to reward. This is in contrast to the devaluation of effortful options in effort-based decision making discussed above (Lopez-Gamundi et al., 2021). Specifically, increased effort expectations lead to increased preparatory activity in ACC (Krebs et al., 2012; Vassena et al., 2014) and the ventral tegmental area/substantia nigra complex (VTA/SN; Krebs et al., 2012), especially when effort is associated with higher reward. This aligns with the postulation that the ACC signals the need for control based on the expected value of control (Shenhav et al., 2016), as well as with the RML Boost module operationalization of dorsal ACC function (Silvetti et al., 2018). In addition to enhanced activity in ACC for both higher effort reward and demand, (ventral) striatum has been implicated in both reward- and demand-based preparation (Suzuki et al., 2021; Vassena et al., 2014). Interestingly, even in the absence of explicit effort-based decisions, when an effort cue is not immediately followed by effort exertion, VS, VTA/SN, and ACC can be sensitive to effort-discounted reward value (Croxson et al., 2009).

VTA/SN is the main source of dopaminergic innervation across the brain. Dopamine has a role in effort modulation, with higher dopamine synthesis capacity predicting higher motivation to exert effort, and dopaminergic stimulants increasing tolerance toward effort, especially in humans with low dopamine synthesis capacity (Westbrook et al., 2020). Indeed, dopamine functioning within the valuation network, that is, striatum and vmPFC, predicts the willingness to exert effort for rewards (Treadway et al., 2012). Complementary to the dopamine system, optimization of costly behaviors also involves the neurotransmitter noradrenaline and its main source region, the locus coeruleus (LC). LC activity is relevant for attention, arousal, stress, and goal-directed behavior (for a review see Poe et al. (2020)). Due to its small size and location, insights into the role of the LC in effort processing are mostly based on single-unit recordings of non-human primates or relying on pupil size as a physiological proxy of LC-noradrenaline activity (cf. e.g. Alnæs et al., 2014). These studies have found that LC activity scales with the level of exerted effort during effort initiation and exertion (Bornert & Bouret, 2021; Varazzani et al., 2015). LC activity has additionally been shown to increase with reward magnitude at the onset of a reward predicting cue (Bouret & Richmond, 2015; Varazzani et al., 2015), while reversely at action preparation weakening more for higher rewards (Bouret & Richmond, 2015; Bouret et al., 2012). Taken together, the noradrenergic system seems to have an energizing influence similar to the dopaminergic midbrain. However, where the dopaminergic system is involved in value computation and energizing rewarded actions, the noradrenergic system seems to support effort allocation in general and to provide an additional boost for actions with low or no external incentive value.

Here, we sought to elucidate the role of most prominent structures related to cognitive effort and reward processing by separating effort evaluation, effort preparation, and effort investment. We developed a new paradigm that features two successive cues preceding a counting Stroop task: first, an evaluation cue that informs participants of the reward and effort demand of the upcoming trial, followed by a separate preparation cue that prompts participants to simply prepare for the upcoming task. The analyses focused on preregistered regions of interest, which have been implicated in both effort evaluation and preparation: VS, ACC, VTA/SN, and LC. Additionally, vmPFC activity was investigated in an exploratory analysis due to its role within the value-signaling network. We expected that VS and vmPFC activity would reflect effort discounting during evaluation, where the incentive value is discounted by the cost of effort (Botvinick et al., 2009; Westbrook et al., 2019). During preparation, effort exertion is imminent and should be prepared for based on the value encoded in the evaluation phase. Based on prior neuroimaging studies on effort preparation (Krebs et al., 2012; Vassena et al., 2014), we hypothesized that ACC would promote preparation especially for trials that feature high-effort demand and high reward. While the dopaminergic VTA/SN may respond similarly to ACC (Boehler et al., 2011; Bromberg-Martin et al., 2010; Krebs et al., 2012), the LC might be more sensitive to expected low-reward effort during preparation (Bouret & Richmond, 2015; Bouret et al., 2012). In exploratory analyses, we also assessed activity modulations during effort exertion (the Stroop task) and outcome evaluation (the feedback phase). Further, in addition to hemodynamic activity, pupil size was tracked throughout the experiment as a physiological measure of effort exertion (van der Wel & van Steenbergen, 2018), and dynamic attention allocation during cue processing (Boehler et al., 2011; Unsworth et al., 2022).

## 2 Methods

### 2.1 Participants

The experiment and analyses were preregistered on Open Science Framework (https://osf.io/rzvxk). Forty-five participants (24 women) were recruited via a Ghent University participant recruitment platform (Sona Systems, https://www.sona-systems.com) and a Facebook group (https://www.facebook.com/groups/expsy). The participants were between the ages of 18 and 41 years, with a median age of 23 years. The study was approved by the ethics committee of the Ghent University Hospital. All participants were right handed and reported normal or corrected-to-normal vision and no history of psychiatric or neurological disorders. Participants earned 41.25 euros for their participation and an additional bonus dependent on their performance. Mean bonus was 7.40 euros (SD = 3.55). The session lasted approximately 95 minutes, including 10 minutes for safety briefing and instructions, 75 minutes of scanning time, and 10 minutes for questionnaire administration. The first participant was excluded from the analysis due to a technical issue with a button box in the scanner. Two participants were excluded due to lack of native Dutch skill, having completed an English version that we excluded from the dataset for consistency. One participant was excluded due to closing their eyes during cue presentations, almost falling asleep, and having poor task performance. The final sample consisted of 41 participants (median age = 23 years, SD = 4.7, 23 [56%] women). Before the experiment, all participants gave their written informed consent.

### 2.2 Stimuli and procedure

The experiment was programmed with the Python toolbox PsychoPy (Peirce et al., 2019). To ensure stable luminance for pupillometry, everything in the experiment was presented on a light gray background, with the majority of stimuli being darker than that. See Figure 1 for a schematic representation of the trial structure. In the experiment, participants completed a counting Stroop task, where they were asked to indicate the number of words they see on the screen while ignoring the meaning of the word (Bush et al., 2006). Participants responded via two two-button Cedrus boxes, one for each hand. The demand of the task was a function of congruency: in an easy trial, the meaning of the word(s) corresponded to the number of words on screen, while in a hard trial, the meaning and the number of words did not match.

**Fig. 1.**
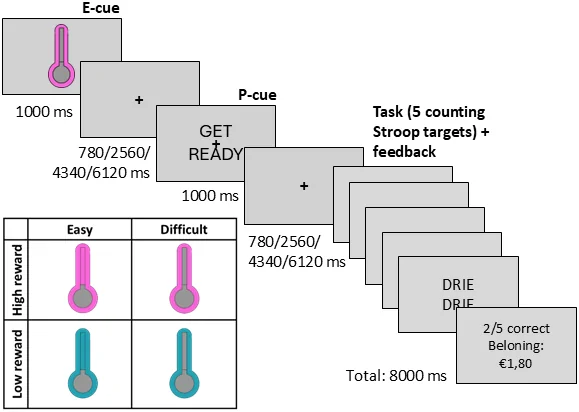
Trial structure. *Note*. Each trial consisted of an evaluation cue (e-cue), a preparation cue (p-cue), five sequential counting Stroop targets each presented for 800 ms followed by 400 ms of blank screen, and trial-wise feedback.

Each trial run comprised an evaluation cue (e-cue), a preparation cue (p-cue), and five successive Stroop targets, ending with a feedback screen with accuracy of the trial run and the corresponding reward sum associated with the correct responses. A sequence of targets was used instead of a single target as our previous work has shown a “miniblock” cuing structure to enhance effort cuing effects (Kukkonen et al., 2025). The stimulus onset asynchronies of e-cue to p-cue and p-cue to first target followed pseudo-exponential distributions, with durations of 1780 ms (1 TR; 71.4% of trials within a block), 3560 ms (2 TRs; 14.3%), 5340 ms (3 TRs; 10.7%), and 7120 ms (4 TRs; 3.6%; *M* = 2606 ms). The inter-trial interval was similarly distributed but was sampled from a range of 2000 to 6500 ms. This stimulus timing approach was adopted to increase statistical sensitivity to BOLD responses to each of the events of interest (Hinrichs et al., 2000). The evaluation cues were four thermometer symbols that could be either of cyan or pink and have either high or low reading. The cue colors were isoluminant to minimize luminance effects on the pupil response. Reward and demand conditions were either indicated by color, with color mappings counterbalanced (high reward/demand = cyan or pink), or by thermometer reading (high reward/demand = high reading, low reward/demand = low reading). Hence, the e-cue color and reading meanings remained stable for each individual but were counterbalanced across four groups. E-cues were followed by p-cue with the text “GET READY” in all conditions. After this p-cue, participants responded to five sequential counting Stroop targets.

Participants were first asked to read and sign an informed consent form, and a screening form to ensure participant eligibility for MRI scanning (e.g., not pregnant, no metal fragments or medical implants in the body). They were then familiarized outside the scanner with the counting Stroop task, the response boxes, and the cue meanings. In the scanner, the participants were asked to show which button to press for each target type (one to four words) to ensure their understanding of the instructions. The task instructions and cue information were displayed again on the screen before the experimental procedure began. The trials were divided into 6 blocks (one scanning run per block) of 28 trials, with 168 trials (42 per condition) in total. The four conditions were presented within each block equal number of times, and in a random order. After each block, participants were informed how many blocks they had completed and could take a short break. The participants finished the breaks by telling the experimenter via the intercom that they were ready to resume the experiment and move on to the next block.

After completing the task in the scanner, participants filled out a post-scan checklist to record any possible adverse effects participants experienced during scanning, such as claustrophobia. Participants were then asked to fill out four questionnaires, namely the Behavioral Inhibition System and Behavioral Activation System Questionnaire (BIS-BAS; Carver & White, 1994), Need For Cognition (NFC, short version; Cacioppo et al., 1984), the General Self-Efficacy Scale (GSE; Schwarzer & Jerusalem, 1995), and the Adult ADHD Self-Report Screening Scale for DSM-5 (ASRS-5; Ustun et al., 2017). BIS-BAS was administered to probe individual differences in reward responsiveness, that is, the sensitivity to positive reinforcement. NFC measures the individual propensity to engage in and enjoy cognitive challenges, hypothetically increasing the intrinsic value of mental effort. GSE measures the belief in one’s own ability to respond to challenges, a trait that might affect how cued task information is used. Last, non-clinical ADHD traits were measured to explore the influence of possible attention-related challenges on effort processing.

### 2.3 Behavioral analyses

Participants’ performance was analyzed with R 4.5.0 (R Core Team, 2025) in conjunction with RStudio 2024.12.1. The “lme4” package (Bates et al., 2015) was used to fit RT and accuracy data with (generalized) linear mixed-effects models, including the fixed effects of reward, demand, and target position within a trial and a maximal converging random effects structure. RT data were modeled with an inverse Gaussian distribution coupled with a log link function^1^ to account for the positive skew typical of RT data (Lo & Andrews, 2015). No random effects beyond a random intercept per participant converged. For accuracy, a random intercept and random slope for demand and reward interaction, and a random slope for target number per participant were included. P-values were calculated using the “car” package (Fox et al., 2019) Wald test. All participants’ accuracies within the final sample were above preregistered exclusion criterion of 55% accuracy. Trials that were responded to faster than 200 ms were excluded, as well as trials that were responded to slower than 2.5 standard deviations (SD) above the participant- and condition-wise mean. This led to the exclusion of 698 trials in total, leaving between 7686 and 8083 correct trials per each condition in the RT analyses (incorrect trials are excluded). As such, based on a power simulation by Brysbaert and Stevens (2018), the experiment was highly powered to detect small effects (Cohen’s d = 0.2).

As an exploratory analysis, scores from the questionnaires were added into models of RT and accuracy as continuous predictor variables to probe the moderating influence of inter-individual trait variability in effort cuing effects. In these analyses, the target position was not included in the models to decrease model complexity and ensure model convergence.

### 2.4 fMRI acquisition and preprocessing

A 3 Tesla Prisma scanner system (Siemens) with a 64-channel head coil was used to acquire 176 T1-weighted anatomical MPRAGE axial slices (acquisition matrix 256 × 256, voxel size 1 mm^3^, flip angle 9°), EPI sequence T2*-weighted MRI transverse whole-brain echo planar images (EPIs; 64 × 64, TR/TE/flip angle = 1780 ms/27 ms/66°) with BOLD contrast, and double echo field map (TR/TE/flip angle = 528 ms/4.92 ms/60°) before functional scans. To acquire participant-specific localizer scans for the LC, 192 T1-weighted turbo spin echo slices (T1-TSE; TR/TE/flip angle 1/flip angle 2 = 559 ms/9.8 ms/70°/180°, acquisition time 9:51 minutes) with voxel size 0.5 × 0.5 × 3.0 mm were acquired after the functional scans. In addition, a T2-weighted scan (TR/TE = 3200 ms/408 ms, acquisition time 5:09) with voxel size 0.4 × 0.4 × 0.9 mm was acquired to enable high-resolution localization of the midbrain regions (VTA/SN).

All fMRI data pre-processing was carried out with SPM12 (https://www.fil.ion.ucl.ac.uk/spm/software/spm12/) and MATLAB (version R2021 9.11.0, The MathWorks). The raw DICOM images were converted to NIfTI format. The 3D images constituting the functional scans were further converted to a single 4D time series. After realigning the functional scans to the mean of all functional scans per participant, the participant-specific T1-weighted anatomical scan was co-registered with the mean functional scan. The co-registration between the mean functional scan and the anatomical scan was inspected visually. The co-registered anatomical scan was then segmented into tissue probabilities based on tissue priors provided by SPM. The resulting deformation field was used to inform normalization of the functional scans into MNI152 standard space. Functional scans used for whole-brain analyses were resliced into 3.5 mm voxel resolution, whereas 2.5 mm^3^ voxel resolution was used for region-of-interest (ROI) analyses. The latter remained unsmoothed, while the former were smoothed with a 6-mm^3^ FWHM Gaussian kernel. T2 scans were co-registered to the mean functional scan and T1-TSE scans were co-registered to the anatomical scan due to its higher resolution. Both were normalized into the standard MNI space.

### 2.5 fMRI data analyses

#### 2.5.1 First level model

Participant-specific BOLD responses for the onsets of the e-cue and p-cue, the onset of the first target, and the onset of trial-wise feedback were modeled as stick functions. The stick functions were convolved with the canonical hemodynamic response function and entered into a general linear model. Only the onsets from trials where the first target was responded to correctly were modeled as regressors of interest.

For each block (six), each participant, and each trial event (e-cue, p-cue, target, feedback), four contrast images associated with each condition (*reward* × *demand*) were created. This led to a total of 17 regressors, including a regressor of no interest for trials in which the first target was responded to incorrectly. Six motion regressors and their time derivatives were added as subject- and block-specific nuisance regressors, and two constant regressors modeled block-specific effects. Volume-wise movement outliers detected using the ART toolbox (nitrc.org/projects/artifact_detect/) were modeled as dummy variables. These were volumes with frame-wise displacement of more than 1.25 mm (half a voxel), or signal intensity change larger than 3 standard deviations (ART default). Regressed volumes per participant varied between 20 and 219, with mean of 73.60 volumes regressed out. Additionally, runs with more than 2.5 mm of motion (voxel) were excluded. This led to exclusion of five runs in total, with a maximum of two runs excluded per participant. A high-pass filter with a threshold of 128 seconds was used to remove low-frequency noise associated with scanner drift. For completeness, a whole-brain analysis is included in the Supplementary Materials (see Supplementary Table S1 for an overview of significant clusters across the respective phases).

#### 2.5.2 ROI analysis

To probe activity modulations of brain regions across the two effort phases, four brain regions commonly shown to subserve reward- and effort-related processes were taken to pre-registered ROI analyses: ACC, SN/VTA, LC, and VS. Activity within a vmPFC ROI was investigated in an exploratory analysis that was not preregistered.

The subject-specific masks for the VTA/SN complex and LC were drawn with MRIcron (https://www.nitrc.org/projects/mricron) onto the normalized T2 and T1-TSE scans based on previous work (Hall et al., 2024; Krebs et al., 2011; Fig. 2; see Supplementary Fig. S1 for more detailed illustration of LC mask). The VS mask was a modified version of an anatomical mask created for a previous study (Asci et al., 2019). This mask was edited to fit the averaged functional scans of the present study that were affected by some data loss in the ventral parts. For the ACC, a spherical mask of 5-mm radius was used with center coordinates based on Krebs et al. (2012), specifically dorsal ACC or anterior midcingulate cortex. To explore how far activity in this ROI is related to general salience, the manipulation effects were compared with those of a more medial/ventral dACC region (5-mm radius sphere, left-lateralized), which is considered a canonical salience network node (Seeley et al., 2007). The vmPFC mask was identical to the sphere-based ROI used in a meta-analysis of neuroimaging studies on effort-based valuation, with radius of 6-mm based on previous work^2^ (Lopez-Gamundi et al., 2021). All masks were finally resliced to the functional scan resolution. The beta values extracted from the ROIs were analyzed on RStudio with separate rANOVAs for each trial event (e-cue, p-cue, target, feedback), all including the factors of *reward* (high versus low) and *demand* (easy versus hard). Significant interactions were disentangled with post-hoc t-tests. The regions themselves, as well as predicted modulations and the applied analyses, have been specified a priori. Hence, the researcher degrees of freedom that motivate family-wise correction were constrained by preregistration (Gelman & Loken, 2013). We, therefore, report uncorrected thresholds as our primary inference, but tests that survive Holm–Bonferroni correction are also reported for transparency.

**Fig. 2.**
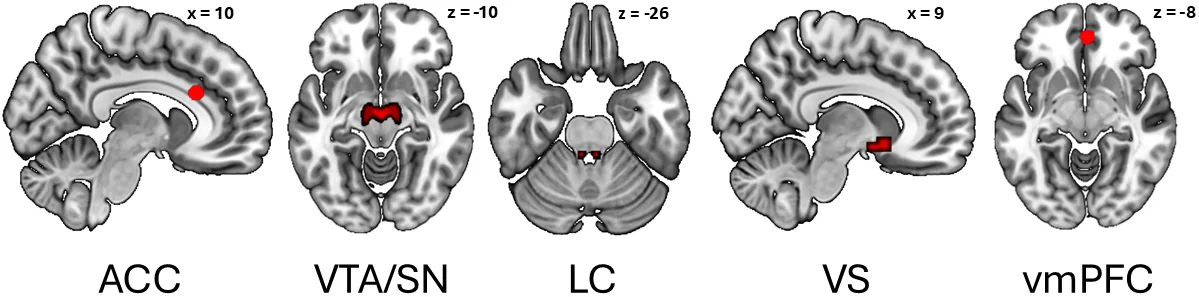
ROI masks. *Note*. ACC = anterior cingulate cortex, VTA/SN = ventral tegmental area/substantia nigra complex, LC = locus coeruleus, VS = ventral striatum, vmPFC = ventromedial prefrontal cortex.

### 2.6 Pupillometry recording, preprocessing, and analysis

To include a robust, objective measure of cognitive effort, pupil size was recorded with a long-range Eyelink 1000+ eye-tracking system (SR Research; Ottawa, Canada; www.sr-research.com) concurrently with fMRI data acquisition. At the beginning of each block, prior to recording, the position of the eye was calibrated with a 9-point calibration grid. After the calibration procedure, position and size of the left pupil were recorded with a sampling rate of 500 Hz throughout the block. Event-locked trigger messages with information of trial phase (e-cue, p-cue, task) and condition (high vs. low reward, easy vs. hard) were sent from PsychoPy and recorded with the eye-tracking data.

Preprocessing of the pupil data was carried out with Python package “pypillometry” (Mittner, 2020). The onsets and offsets of blinks were determined based on pupil signal values of 0 and the velocity of pupil size change. The pupil signal was low-pass filtered with a Butterworth filter of 4 Hz, attenuating any fluctuations in pupil size faster than 4 Hz, and blinks within 100 ms of each other were merged. Last, the blinks were interpolated (i.e., pupil signal was reconstructed) with an algorithm described in Mathôt et al. (2013). Out of the final sample (*N* = 41), the pupil of five participants was not tracked due to eyeglasses that prevented detection of the pupil. Further, 16 participants’ pupil data for all 6 blocks were excluded from the analyses due to excessive signal loss (more than 50% of signal was missing per block) or excessive blinking (more than 30 blinks per minute within block). Subsequently, pupil analyses were based on 20 participants, excluding 31 blocks with low-quality pupil. Hence, the somewhat challenging context of the MR scanning environment made it so that we could only analyze the pupil data of a subsample, thereby limiting the power of the respective analyses.

Statistical analyses of the pupil response were carried out in R. E-cue- and p-cue-locked pupil responses were modeled separately. Pupil signal in both cue phases was baseline-corrected trial-wise by subtracting the mean pupil size within 400 ms before the cue onset from all the pupil size measurements of that event, using separate baseline periods for the e-cue and the p-cue stages. Pupil signal of interest was defined as beginning from 480 ms after cue onset and ending at the end of the shortest and most common SOA period, 1780 ms. Pupil size was analyzed as averaged bins of 50 ms for evaluation and preparation phase pupil analyses, with bin added as a continuous predictor variable into the LMM. Based on visual inspection of the pupil size shape across the bins, evaluation, preparation, and task phase bins were modeled as cubic, quadratic, and linear predictor variables, respectively. For the analysis of task pupil size (five Stroop targets), pupil size was averaged into bins of 150 ms for computational efficiency. As in behavioral analyses, the main effects of reward and demand were tested, as well as their interaction. Linear mixed models were used to model the pupil signal, with a maximal converging random effects structure. Evaluation phase data were modeled with a random intercept and a random slope of demand per participant. Both allocation and task phase pupil signal models included a random intercept, a random slope of interaction between reward and demand, and a random slope of bins (time within phase) per participant.

## 3 Results

### 3.1 Behavioral results

RTs were influenced by *reward* (*X^2^*[1] = 1.90, *p* < .001), *demand* (*X^2^*[1] = 3.57, *p* < .001), and *target order* (*X^2^*[1] = 2.81, *p* < .001; Fig. 3). As expected, pairwise comparisons revealed RTs to be faster in high- versus low-reward condition (*b* = -0.01, SE = 0.002, *z* = -5.75), and in easy versus hard condition (*b* = -0.08, SE = 0.002, *z* = -47.97), and decreased with each successive target. Additionally, *reward* and *target order* interacted (*X^2^*[1] = 6.43, *p* < .05), as the reward-related difference in RTs was significant at the beginning of the trial but disappeared toward the end (first target: *b* = -0.01, SE = 0.003, *z* = -4.78, *p* < .001; final target: *b* = -0.003, SE = 0.003, *z* = -0.85, *p* = .39). *Demand* had an effect on accuracy (*X^2^*[1] = 40.05, *p* < .001), as accuracy was higher in easy than in hard condition (*b* = 0.83, SE = 0.08, *z* = 10.26). *Reward* did not have an effect on accuracy on its own (*X^2^*[1] = 0.04, *p* = .83), but a three-way interaction *reward* × *demand* × *target order* was marginally significant (*X^2^*[1] = 3.64, *p* = .056), suggesting high reward was associated with better accuracy at the end of a sequence of hard targets within a trial run.

**Fig. 3.**
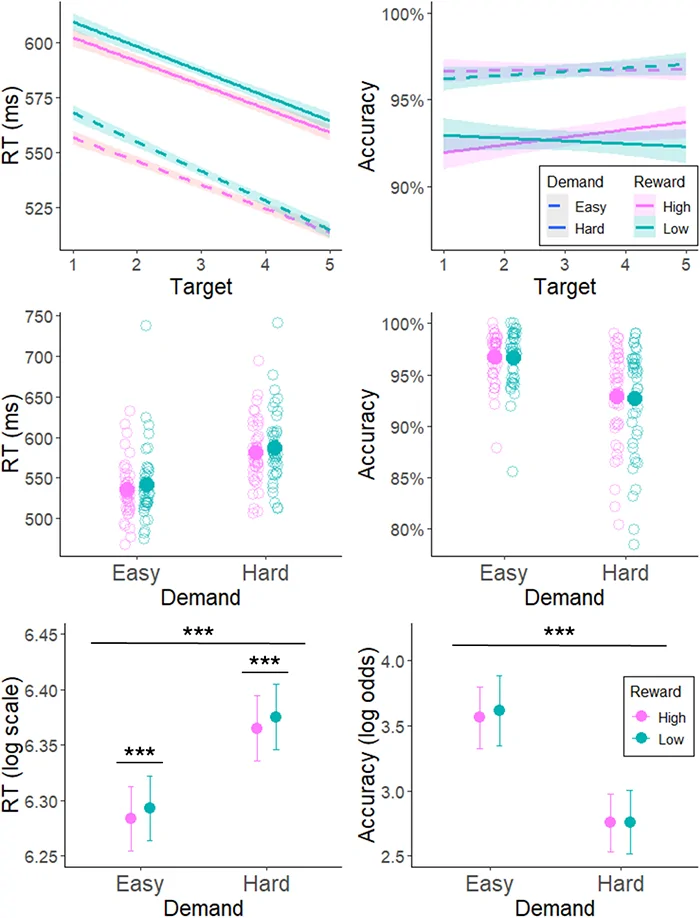
Task performance. *Note*. Linear fit of RT (top left panel) and accuracy (top right panel) across five targets within a trial. Shaded area represents the standard error of the linear trend. Raw RT (middle left panel) and accuracy (middle right panel): hollow points represent participant-wise means. Solid points represent condition-wise means across participants. Modeled RT (bottom left panel) and accuracy (bottom right panel) per condition. Error bars in the bottom panel plots represent the standard error of the estimated marginal means. ***p < .001.

Additionally, the moderating effects of traits on reward effects in RT were probed in exploratory analyses. An interaction between NFC score and reward was found in RTs (*X^2^*[1] = 9.65, *p* < .01). High (+1 standard deviation from mean) and intermediate (mean) levels of affinity for cognitive challenges predicted faster RTs in high-reward targets (high NFC: *b* = -0.02, SE = 0.002, *z* = -7.46, *p* < .001; intermediate NFC: *b* = -0.01, SE = 0.002, *z* = -5.83, *p* < .001), whereas this effect of reward was not significant in participants reporting low levels of NFC (*b* = -0.002, SE = 0.002, *z* = -0.74, *p* = .46). BAS reward responsiveness, that is, sensitivity to positive reinforcement, had a marginal effect on RTs (*X^2^*[1] = 3.79, *p* = .05), as higher reward responsiveness was associated with faster RTs in general. Neither self-efficacy as measured by GSE or ASRS-5 score had any significant effects on RTs. Effects of individual difference scores in accuracy are reported in Supplementary Materials^3^.

### 3.2 ROI results

*Reward* × *demand* rANOVAs of beta values in each of the ROIs were run separately for each of the effort stages, namely the evaluation stage, the preparation stage, the target stage, and the feedback stage. No significant effects were found in the LC at any of these stages and will, therefore, not be further discussed here (see Supplementary Fig. S3 for further details). In addition to the analyses reported here, exploratory models with additional predictor variable of *experiment half* were run to test the effect of time on the manipulation effects. Further, to investigate the manipulation effects within the wider control and reward networks, exploratory analyses were conducted within putamen and caudate (5-mm radius sphere ROIs, MNI center coordinates -19, 9, 2; 17, 9, -2; -10, 9, 2; and 10, 9, 2; respectively). The results of these analyses are reported in the Supplementary Materials.

In the evaluation phase, no significant modulations based on reward or demand prospect were found in ACC or VTA/SN (Fig. 4). However, both the VS and vmPFC showed a significant effect of reward (VS: *X^2^*[1] = 4.71, *p* < .05; vmPFC: *X^2^*[1] = 4.13, *p* < .05), with higher activity for high- than for low-reward e-cues. vmPFC revealed an additional sensitivity to effort (*X^2^*[1] = 4.72, *p* < .05), with higher activity in response to easy than to hard e-cues. A marginal interaction suggested a reward effect specifically in the easy condition (*X^2^*[1] = 2.95, *p* = .09).

**Fig. 4.**
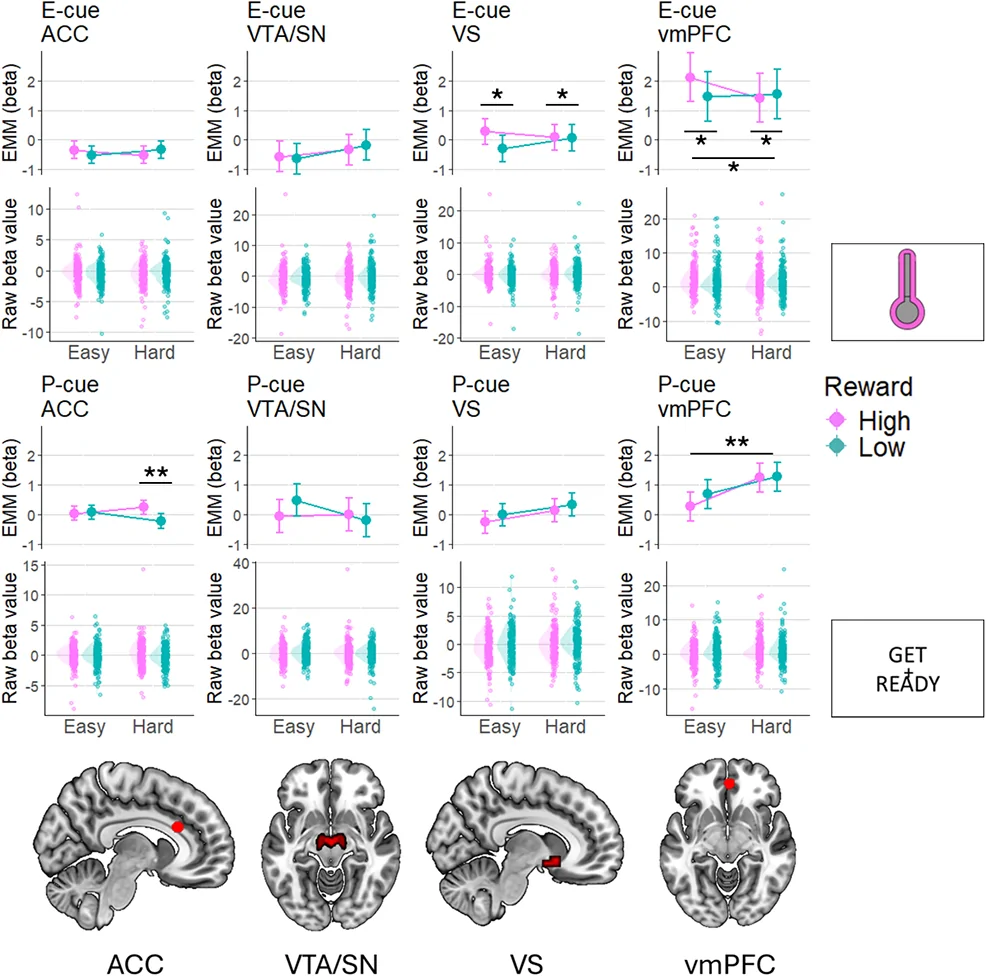
Beta values associated with each region of interest and trial phase. *Note*. Two upper panels: hemodynamic activity within ROIs during evaluation (e-cue). Two lower panels: hemodynamic activity during preparation (p-cue). In both cases, higher panel depicts the estimated marginal means of the beta values, lower panel depicts the raw beta values and the raw data distribution. Error bars represent the estimated standard error of the estimated marginal mean. EMM: estimated marginal mean; ACC: anterior cingulate cortex; VTA/SN: ventral tegmental area/substantia nigra complex; VS = ventral striatum; vmPFC = ventromedial prefrontal cortex. *p < .05; **p < .01.

In the preparation phase, participants were prompted by a p-cue to prepare for the upcoming target sequence, with no reward or demand information on the screen anymore. ACC activity in this phase was characterized by a significant *reward* × *demand* interaction (*X^2^*[1] = 5.69, *p* < .05). This interaction reflected an activity increase for high compared with low reward when a hard target was expected (*b* = 0.47, SE = 0.15, *t* = 3.10, *p* < .01) that was not significant on easy trials (*b* = -0.04, SE = 0.15, *t* = -0.28, *p* = .78). In contrast, the more medial and ventral ACC ROI centered on salience network node revealed no significant effects, providing evidence against the possibility that the preparation effects within our ACC ROI reflect a general salience response. No significant activity modulations based on reward or demand prospect were revealed in VS or VTA/SN during the preparation phase. Activity in vmPFC (*X^2^*[1] = 9.49, *p* < .01) was significantly affected by demand prospect, reversing the demand effect from e-cue with higher activity in preparation for hard targets than for easy targets. After Holm–Bonferroni correction of the evaluation and preparation phase tests of each ROI, only the vmPFC effort effect in the preparation phase survived as statistically significant.

We also explored activity in these ROIs during the effort exertion stage (Fig. 5). As expected, ACC was more active during engagement in hard (incongruent) targets than in easy targets (*X^2^*[1] = 6.37, *p* < .05). VTA/SN displayed higher activity when exerting effort for a high reward (*X^2^*[1] = 5.21, *p* < .05). In VS, no significant effects were observed in the target phase. Finally, easy targets were associated with stronger activity in vmPFC than hard targets (*X^2^*[1] = 12.45, *p* < .001), likely reflecting the role of vmPFC within the default mode network (Raichle, 2015).

**Fig. 5.**
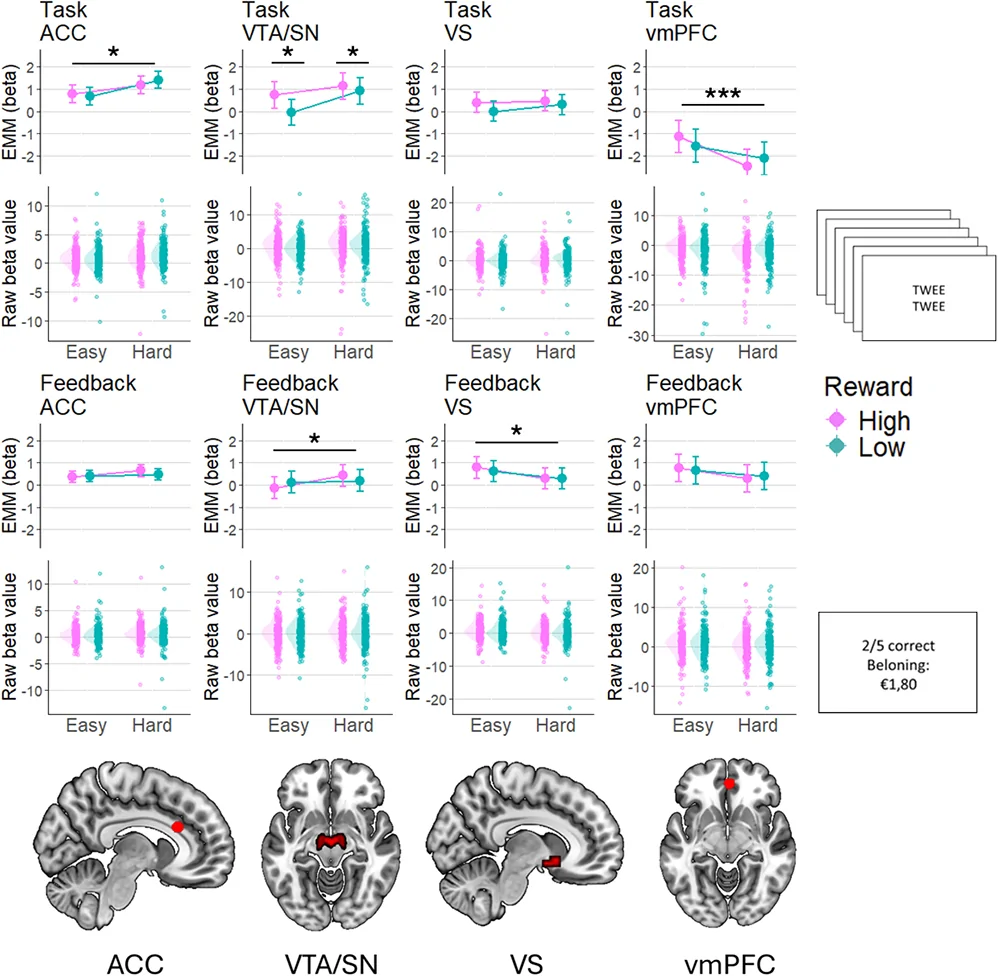
Beta values of each ROI within task and feedback phase. *Note*. Two upper panels: hemodynamic activity within the ROIs during the five Stroop targets (task). Two lower panels: hemodynamic activity during feedback. In both cases, higher panel depicts the estimated marginal means of the beta values, and lower panel depicts the raw beta values. Error bars represent the estimated standard error of the estimated marginal mean. EMM: estimated marginal mean; ACC: anterior cingulate cortex; VTA/SN: ventral tegmental area/substantia nigra complex; VS = ventral striatum; vmPFC = ventromedial prefrontal cortex. *p < .05; ***p < .001.

Lastly, ROI activity during trial-wise feedback was investigated without any distinction concerning outcome. As with task-related activity, no hypotheses were preregistered regarding these analyses. Both VTA/SN (*X^2^*[1] = 4.07, *p* < .05) and VS (*X^2^*[1] = 4.07, *p* < .05) responded to the demand condition when processing feedback but revealed opposite effects: feedback processing after easy trials was associated with more VS activity than after hard trials, while VTA/SN responded more to feedback after hard trials. None of the other ROIs revealed significant effects of reward or demand during feedback presentation. After Holm–Bonferroni correction of the task and feedback phase tests of each ROI, only the vmPFC effort effect in the task phase survived as statistically significant.

### 3.3 Pupil results

Manipulation effects on pupil size were examined in a subsample (*n* = 20) as an additional physiological index of attention, arousal, and effort. Here, we expected larger pupil to reflect increased attention or arousal in the evaluation and preparation phase, and increased effort exertion in the task phase. Pupil size across evaluation phase, preparation phase, and task is shown in Figure 6. A significant effect of *bin* (*X^2^*[2] = 347.45, *p* < .001) indicated that pupil constricted within the evaluation phase (bin 1 – bin 25: *b* = 172, SE = 4.35, *z* = 39.60). An interaction between *reward* and *demand* in the evaluation phase (*X^2^*[1] = 29.84, *p* < .001) showed that pupil constricted less (remained larger) for high reward compared with low reward only in the hard condition (*b* = 31.55, SE = 5.27, *z* = 5.98, *p* < .001; easy condition high–low: *b* = 2.74, SE = 5.27, *z* = 0.52, *p* = .60).

**Fig. 6.**
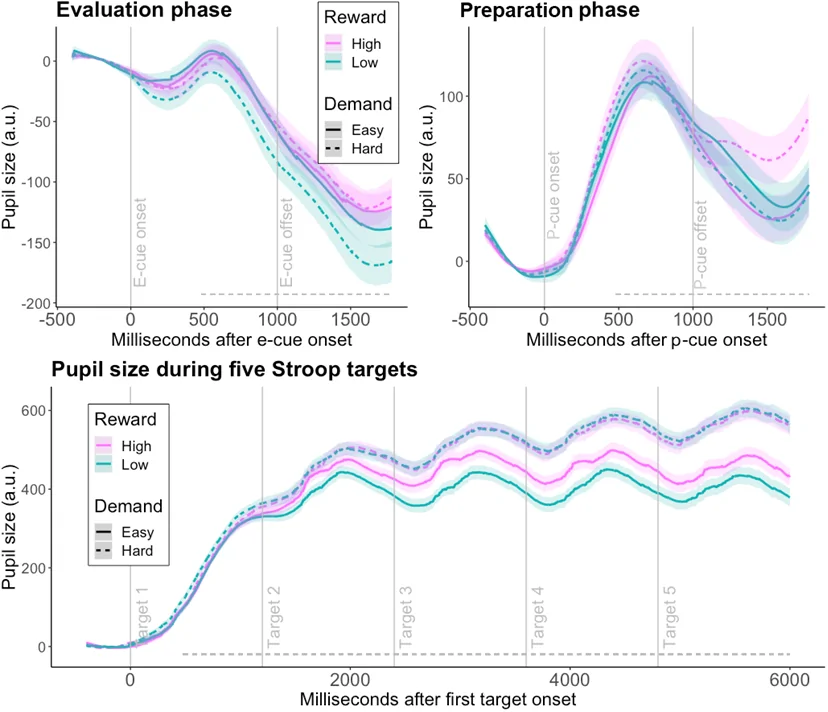
Trace plots of pupil size. *Note*. Trace plot of pupil size from the onset of evaluation cue (top left), preparation cue (top right), and throughout the sequence of five Stroop targets (bottom). Shaded area represents the standard error. Horizontal gray dotted line denotes the timeframe of the analyzed pupil signal. a.u. = arbitrary units.

During the preparation phase, the pupil size was affected by an interaction between *bin*, *reward*, and *demand* (*X^2^*[2] = 11.32, *p* < .01). Inspecting the reward and demand interaction at first and last bin, no simple effect was significant (all *p*’s > .19), but the effect of reward was reverse in hard versus easy condition: in easy condition the larger pupil for high reward decreases its size difference with low reward toward the last bin (first bin: *b* = 17.53, SE = 27.1, *z* = 0.65; last bin: *b* = 4.89, SE = 26.4, *z* = 0.19), while in the hard condition, the pupil size for high reward versus low reward increases (first bin: *b* = 9.35, SE = 41.5, *z* = 0.23; last bin: *b* = 54.41, SE = 41.1, *z* = 1.32).

Testing the effect of our effort manipulation, we also investigated pupil responses during the task, that is, five sequential Stroop targets. *Demand* and *bin* interacted (*X^2^*[1] = 92.43, *p* < .001), as pupil was larger during hard targets, especially toward the end of the target sequence. *Reward* and *bin* also shared a significant interaction (*X^2^*[1] = 14.58, *p* < .001): high-reward targets were associated with a larger pupil toward the end of a trial. Last, a three-way interaction was uncovered between *reward*, *demand*, and *bin* (*X^2^*[1] = 6.53, *p* < .05). Disentangling the interaction revealed that a reward effect in pupil emerged with time when engaging with easy target sequences (first bin: *b* = 7.95, SE = 26.7, *z* = 0.30, *p* = .77; last bin: *b* = 56.65, SE = 26.7, *z* = 2.13, *p* < .05). Reward did not have an effect in hard target sequences (first bin: *b* = -18.74, SE = 27.6, *z* = -0.68, *p* = .50; last bin: *b* = -16.14, SE = 27.6, *z* = -0.59, *p* = .56).

To examine the relationship between pupil size and performance, averaged and z-transformed (within participant) target-wise pupil size was added as a predictor in models of RT and accuracy (*reward* × *demand* × *target order* × *pupil*). A significant effect of *pupil* showed that a larger pupil (+1 SD vs. -1 SD within participant) during task engagement was associated with faster RTs (*X^2^*[1] = 5.54, *p* < .05). A significant interaction between *reward*, *target order*, and *pupil* size (*X^2^*[1] = 4.06, *p* < .05) indicated that larger pupil was associated with faster RTs especially at the beginning of the trial when reward was high (large–small pupil: *b* = -3.62, SE = 0.01, *z* = -2.83, *p* < .01). No significant effects of pupil size were uncovered in accuracy. Performance effects of pupil size during evaluation and preparation were also explored but revealed no significant effects on RTs or accuracy.

## 4 Discussion

In this study, we set out to investigate how the brain supports effort processing in different phases, from evaluation to preparation to effort exertion. While studies on effort-based decision making have shown that effort is encoded as a cost that discounts incentive value, effort prospect can function as a motivator when effort exertion is imminent, rendering effort signals as an investment, or amortized cost. To parse both modes of effort encoding, on each trial two successive cues preceded a cognitive task that participants completed in an fMRI scanner with concurrent pupil tracking. An evaluation cue informed participants of the reward and effort level of the upcoming target sequence, and a following preparation cue prompted participants to prepare for the task. As such, neural activity modulations during the first cue were thought to mainly probe evaluation of reward and effort contingencies, while processing the second cue mainly taps into the realm of preparatory effort. Focusing on neural structures commonly implicated in reward and effort processing, namely ACC, VTA/SN, and VS and vmPFC, we show that these regions display distinct neural activity profiles during different stages of effort processing.

Based on the idea that ACC synthesizes current effort value and control requirements to monitor and adjust control levels accordingly (Shenhav et al., 2013, 2016), activity within this region should signal the net value of effort in the evaluation phase (Croxson et al., 2009), turning the negative signal of effort into a positive one in the preparation phase (Krebs et al., 2012). However, based on a recent meta-analysis, it was more likely that ACC would encode the prospective effort demand level, without representing valuation signals that are encoded within other regions (Lopez-Gamundi et al., 2021). Contrary to both expectations, we found no evidence of ACC involvement in the evaluation phase. In the preparation phase, we found preferential ACC activity in preparation for trials associated with high gain and high effort, replicating prior findings by studies using similar cued effort tasks (Krebs et al., 2012; Vassena et al., 2014). In a meta-analysis by Lopez-Gamundi et al. (2021), ACC activity tracked pre-SMA activity, scaling positively with demand and negatively with effort net value. The lack of ACC response during the evaluation phase aligns with their interpretation that ACC and pre-SMA are not involved in value integration but rather encode the effort demand at hand. During the preparation phase, ACC utilizes reward information to signal heightened need for resource recruitment, reflecting its role as a supervisory hub that integrates value and control signals to mobilize adaptation to the imminent control needs. Taken together with the parallel effects in pupil size, which is used as a proxy for LC activity, these results align closely with the Boost module of the Reinforcement Meta Learner model of dACC function (RML; Silvetti et al., 2018).

While uncovering the expected interaction within ACC in the preparation phase, this study did not find evidence of VTA/SN showing preferential activity for high-reward high-demand trials. Instead of responding to reward or demand in either evaluation or preparation phase, VTA/SN invigorated online effort engagement for high reward. This is at odds with earlier reports of VTA/SN supporting preparation for high effort especially when rewarded (Krebs et al., 2012) but also in the absence of external incentives (Boehler et al., 2011). A recent fMRI study aligns with our results: VTA/SN showed a reward response during rewarded task execution (1000 ms target) but not during encoding reward-predicting cues (Hall et al., 2024). In Hall et al. (2024), like in the present study, the reward cue was separated from task execution by a relatively long interval. In this context, the present results suggest that if task performance is by design disentangled from cue encoding, activity in this region is mostly aligned with action invigoration. Moreover, in contrast to the earlier studies that involved a one-off, fast (30 ms or 100 ms target duration) visual discrimination target in each trial, the trials of the current experiment necessitated solving cognitive conflict across five successive Stroop targets. It is possible that the former type of task demands higher response readiness and visual attention, imbuing the cues with more behavioral relevance: the VTA/SN complex would thus exhibit an alerting response to the salient cues of high demand (and high reward; Bromberg-Martin et al., 2010; Schultz, 1998). SN might also promote earlier motor preparation in these types of tasks (Alexander et al., 1986; Haber, 2014) compared with Stroop that relies more on cognitive evidence accumulation. In sum, our results reflect the well-established role of the dopaminergic midbrain in goal-directed behavior invigoration (Robbins & Everitt, 2007; Schultz, 1998), and suggest that when evaluation and preparation are clearly separated from task execution, VTA/SN activity reflects action invigoration rather than more complex preparatory facilitation like that of ACC. In the feedback phase, VTA/SN neurons activated more after a hard trial (lower success probability), potentially reflecting a reward prediction error (Schultz et al., 1997; Wise, 2005).

VS and vmPFC have been identified as the loci of a domain-general valuation network, informing our hypothesis that these areas might encode the net value of effort during the evaluation phase, translating in this experiment into heightened activity for high-reward, low-effort trials in the evaluation phase. Consistently, activity in both VS and vmPFC during the evaluation phase showed a positive reward effect. Additionally, vmPFC exhibited preferential activity in response to easy versus hard cues in line with the idea that vmPFC encodes effort as a cost similar to delay or risk, decreasing the value of the upcoming trial (Bartra et al., 2013; Lopez-Gamundi et al., 2021; Westbrook et al., 2019). Interestingly, the negative demand response in vmPFC turned into a positive demand response in the preparation phase. It might be that in the absence of choice, vmPFC reflects cognitive regulation to assure appropriate control, possibly in communication with other regions such as ACC. While vmPFC reliably encodes the net value of a chosen option or an expected reward (Bartra et al., 2013; Lopez-Gamundi et al., 2021), the role of vmPFC likely extends to optimization of task state and goal representations (Fine & Hayden, 2022; Froemer et al., 2024; Hayden & Niv, 2021; Moneta et al., 2024; Simoens et al., 2025). It could be that the separation of effort evaluation and preparation phase revealed two response modes within vmPFC that could not be distinguished in experiments with either simultaneous effort valuation and preparation (cued effort) or an active decision phase. Future studies will be necessary to better understand the functional profile of vmPFC in cognitive effort. Overall, none of the ROIs assigned a negative signal to effort in the preparation phase.

The possible contribution of LC to effort processing was also examined in this study as the central structure of the noradrenergic system. Based on previous animal studies, we expected LC to respond preferentially to the reward value of effort in the evaluation phase (Bouret & Richmond, 2015; Varazzani et al., 2015) and signal a compensatory boost in low-reward trials in the preparation phase (Bouret et al., 2012; Bouret & Richmond, 2015). However, no effects relating to our manipulation were found in LC. It is possible that the resolution of our functional scans was not sufficiently high to capture activity related to this narrow structure, with cerebrospinal fluid of the fourth ventricle potentially adding noise to the signal. Similarly, the lack of significant modulations in VTA during evaluation and preparation might be related to suboptimal scanning parameters for small nuclei in the midbrain that are more prone to signal dropout and distortion (Brooks et al., 2013).

Pupil responses to reward and demand manipulation within the evaluation and preparation phase functioned as a complementary measure to fMRI data of how the effort context was processed within each phase. Pupil diameter is a reliable measure of arousal and can thus give indication of the arousal response to evaluation cues, and how attention is maintained or enhanced during the preparation phase dependent on the condition (Schneider et al., 2018). Paralleling reward-driven ROI effects, pupil remained larger in high-reward than in low-reward evaluation phase. In the preparation phase, pupil size seemed to reflect more the preparatory activity within ACC, dilating more in preparation for high-reward, high-effort targets. Previous studies have reported pupil-related task modulation of ACC activity (DiNuzzo et al., 2019; Ebitz & Platt, 2015; Schneider et al., 2018). Schneider et al. (2018) suggested this relationship to be due to ACC-triggered LC activity. LC is argued to subserve modulation of alertness via noradrenergic release (Aston-Jones et al., 1991; Berridge & Waterhouse, 2003), which, in turn, is associated with pupil dilation (Rajkowski et al., 1994). Signals from ACC might thus facilitate upregulation of alertness via the LC-norepinephrine system. During task execution, changes in pupil size are assumed to reflect effort-related processes (da Silva Castanheira et al., 2021; Unsworth et al., 2022; van der Wel & van Steenbergen, 2018) and/or online performance monitoring (Antony et al., 2021; Braem et al., 2015; de Gee et al., 2021; Preuschoff et al., 2011) rather than mere task difficulty. We found some evidence of this, as larger pupil size during task engagement was associated with faster responding to the Stroop targets, replicating earlier findings (Eayrs et al., 2025).

Distinguishing pre-task effort evaluation and preparation within our fMRI study, we uncovered distinct activity patterns across neural structures and across different phases of reward and effort processing. Our results suggest that the valuation network signals the value of effort, including higher value of low effort, at early stages, shifting to effort preparation within control-relevant regions such as ACC, which, in turn, promotes effort allocation preferably for highly rewarded demanding tasks. Indeed, vmPFC, a region that in the evaluation phase encodes a negative effort signal, assigned effort a positive value in the preparation phase, possibly reflecting its role in higher-order goal representation. The dopaminergic midbrain was not involved in the valuation of, or preparation for, effort but signaled the positive value of reward during effort exertion. The findings of the current study advance a more nuanced understanding of how the brain supports different stages of successful cognitive effort and may help clarify cognitive effort difficulties observed in clinical conditions.

## Supplementary Material

Supplementary Material

## Data Availability

The project is pre-registered at the Open Science Framework (OSF) at https://osf.io/rzvxk and data and code are available at https://osf.io/hc8dv

## Footnotes

1 This model gave a warning about having a very large eigenvalue. An LMM with log-transformed RTs was run in addition, giving near identical results to the GLMM specified, with worse conditional and marginal R2. Other GLMMs with different theoretical distributions and link functions also converged with the results.

2 The 6-mm radius was used here based on the vmPFC ROI reported in the meta-analysis by Lopez-Gamundi et al. (2021). For completeness, the vmPFC ROI analysis was additionally carried out with 5-mm radius ROI, corresponding to our other sphere-based ROIs. The results of 5-mm and 6-mm radius converged.

3 In accuracy, GSE effects paralleled NFC effects. An exploratory Spearman method correlation test revealed that NFC and GSE scores shared a moderate positive correlation (*r* = .42, *p* < .001; Supplementary Fig. S2 in Supplementary Materials).
