# Supplementary material for: Distinct roles of anterior cingulate and subcortical regions supporting effort evaluation versus preparation
Source: Imaging Neurosci (Camb). 2026 Oct 30;4:IMAG.a.1417. doi: 10.1162/IMAG.a.1417 (PMC13726102; doi:10.1162/IMAG.a.1417)
Supplement: Supplementary file 1 [file IMAG.a.1417_supp.pdf]

Whole-brain analysis

Whole-brain analyses were carried out for each phase separately: e-cue, a-cue, task (first target), and feedback. Per each phase, main effects of reward and demand were tested, as well as an interaction between the two. First, clusters uncorrected at $p_{voxel} < .0001$ were selected. Significance threshold was defined as whole-brain family-wise error (FWE) corrected $p_{cluster} < .05$.

See Table S1 for regions exhibiting significant manipulation effects. No significant main effects or interactions were observed in the e-cue phase. In the a-cue phase main effect of reward was uncovered in the inferior parietal lobule (Brodmann area 40), specifically exhibiting higher activity when anticipating high reward compared to low reward. Additionally, significantly higher activation for anticipation of hard versus easy targets was found in dorsomedial prefrontal cortex (dmPFC). No significant interactions were found in the a-cue phase.

In addition to e-cue and a-cue phases, manipulation effects in whole-brain activation in the task phase (only first target, excluding incorrect responses) and feedback phase were examined. No significant main effects of reward or interactions were found in task phase, while significant main effects of demand were discovered in widespread regions. Post-hoc t-tests showed that areas activated preferentially during hard versus easy targets included large clusters within the midbrain, ventral prefrontal cortex, parietal-temporal-occipital association area, cerebellum, and less widespread activity around the posterior area of the inferior temporal cortex. In the feedback phase, there were no significant main effects of reward, or interactions between reward and demand, but large clusters exhibiting sensitivity to demand were once again uncovered. Specifically, viewing feedback after completing hard versus easy

trials was associated with increased activity in the anterior cingulate cortex, insula, cerebellum, and supramarginal gyrus.

Table S1.

Regions with significant manipulation effects

Phase	Region (peak)	L/R	*k*	MNI coordinates			*Z*
				x	y	z	
A-cue	*High > Low reward*						
	Inferior parietal lobule	L	143	-34	-35	38	3.87
	Hard > Easy						
	Dorsomedial prefrontal cortex	L	87	-2	49	35	3.85
Task	*Hard > Easy*						
	Midbrain	R	918	8	-18	-10	6.13
	Inferior frontal operculum	L	891	-44	7	28	5.80
	Middle occipital gyrus	L	899	-27	-66	42	5.00
	Cerebellum	L	603	-16	-49	-21	4.34
	Inferior temporal gyrus	L	99	-48	-56	-10	4.01
	Easy > Hard						
	Superior frontal gyrus	R	499	18	42	42	5.02
Feedback	*Hard > Easy*						

Anterior cingulate cortex	R	218	4	35	24	5.74
Insula	R	575	46	21	-7	5.60
Vermis	R	1093	1	-74	-35	5.46
Supramarginal gyrus	R	301	54	-38	42	4.75

Note. Region corresponds to the location of the voxel with peak activity within the cluster. L: left hemisphere; R: right hemisphere; *k*: cluster size; MNI: Montreal Neurological Institute; F value: FWE-corrected cluster-level significance threshold at $p < .05$.

Figure S1. Locus coeruleus mask.

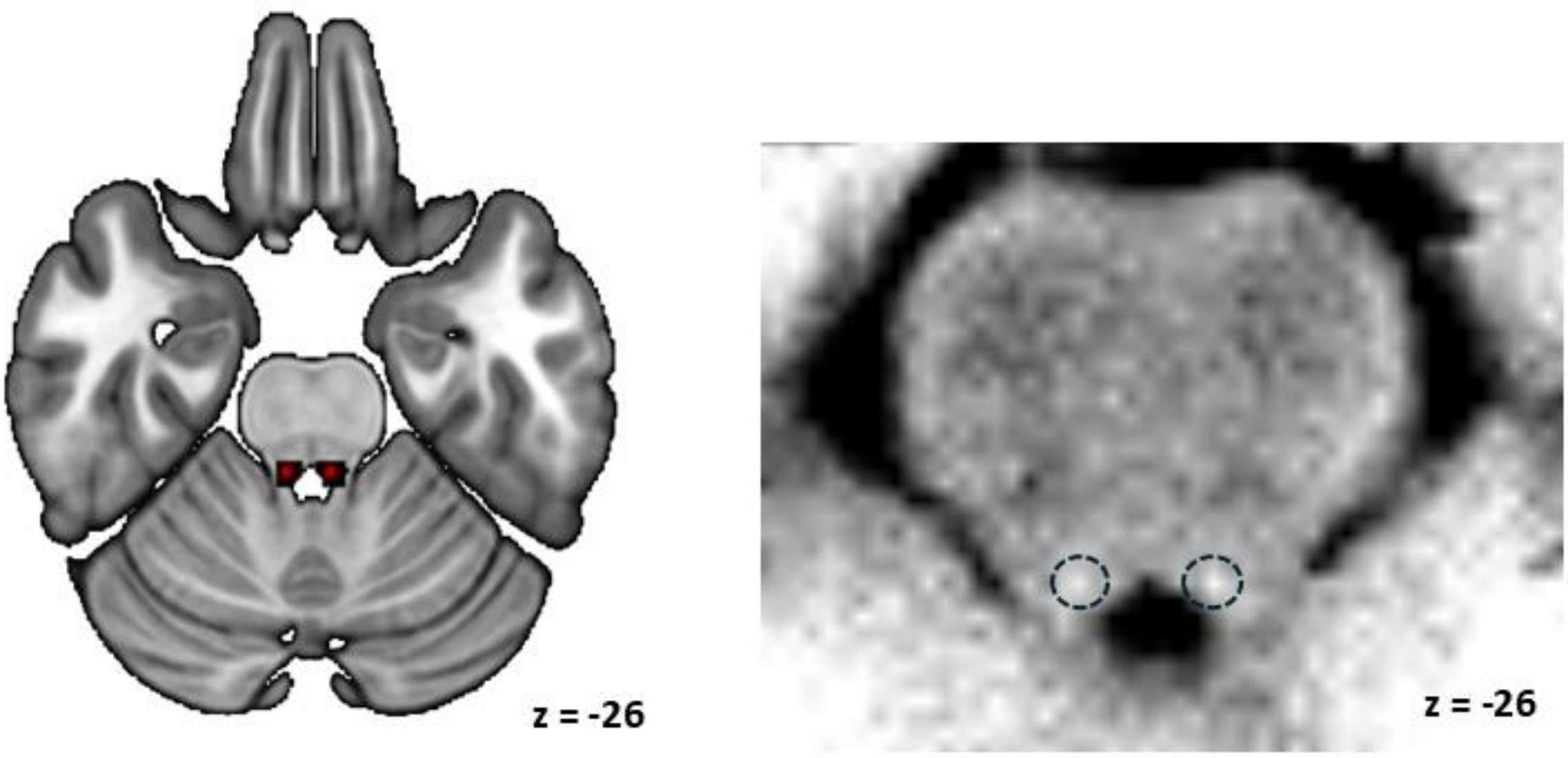

Note. A mean of locus coeruleus masks (left) and locus coeruleus circled on a single-participant T1-TSE scan (right).

Figure S2. Visualization of the correlation between participants' reported Need For Cognition (NFC) and General Self-Efficacy scale (GSE) score.

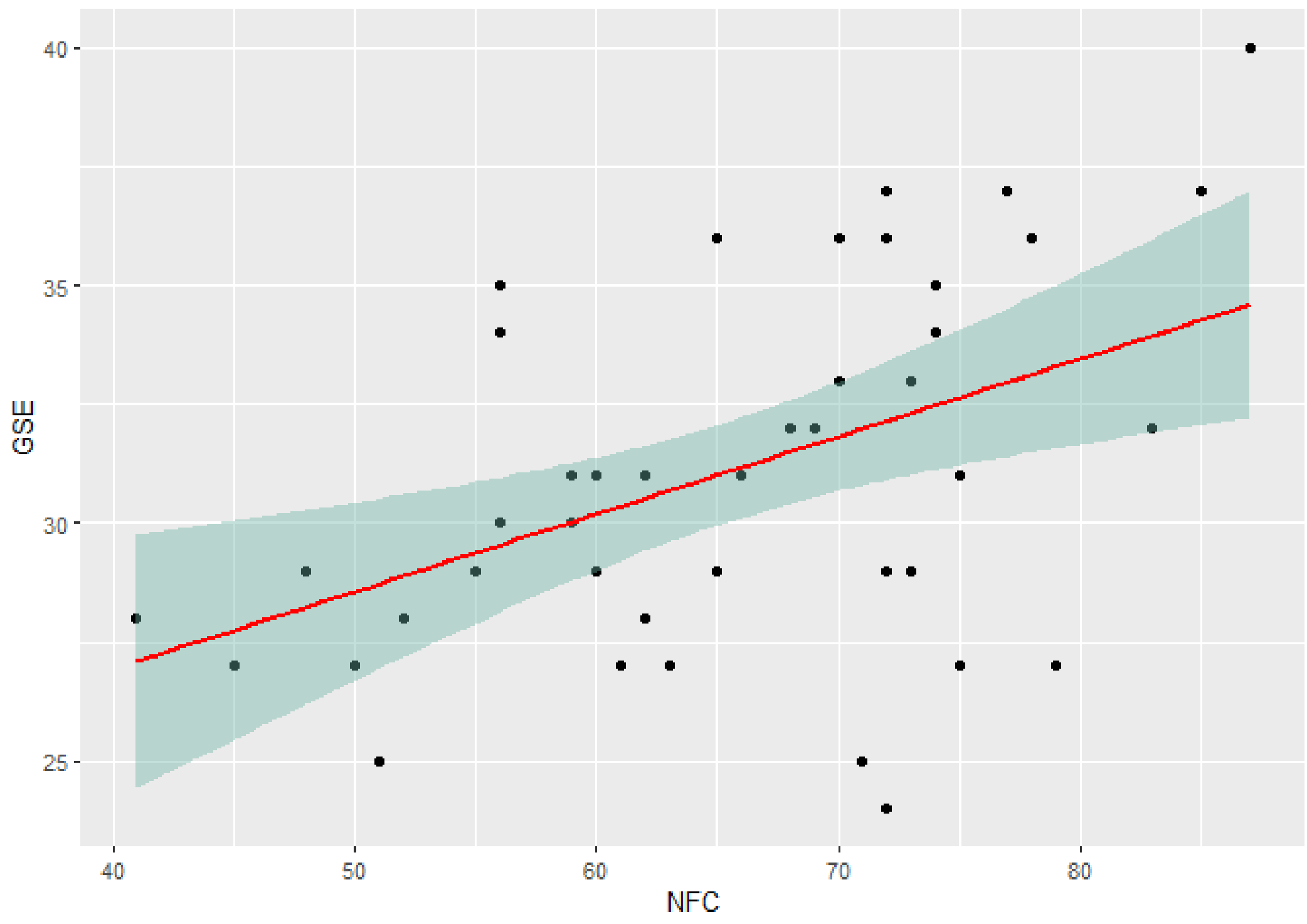

Inter-individual differences in accuracy

In accuracy, both NFC ($X^2[1] = 8.61$, $p < .01$) and self-efficacy (as measured with GSE; $X^2[1] = 10.48$, $p < .01$) interacted with reward and demand in separate three-way interactions. Participants with low NFC responded to high-reward (versus low-reward) easy targets with higher accuracy ($b = 0.25$, SE = 0.12, $z = 2.07$, $p < .05$). Similarly, low self-efficacy was associated with a reward-related accuracy improvement in easy targets ($b = 0.31$, SE = 0.13, $z = 2.38$, $p < .05$), while high self-efficacy was associated with worse accuracy for high reward (versus low reward) easy targets ($b = -0.25$, SE = 0.13, $z = -1.98$, $p < .05$). None of the other measures had significant influence on the accuracy effects.

Figure S3. Locus coeruleus beta values associated with each trial phase.

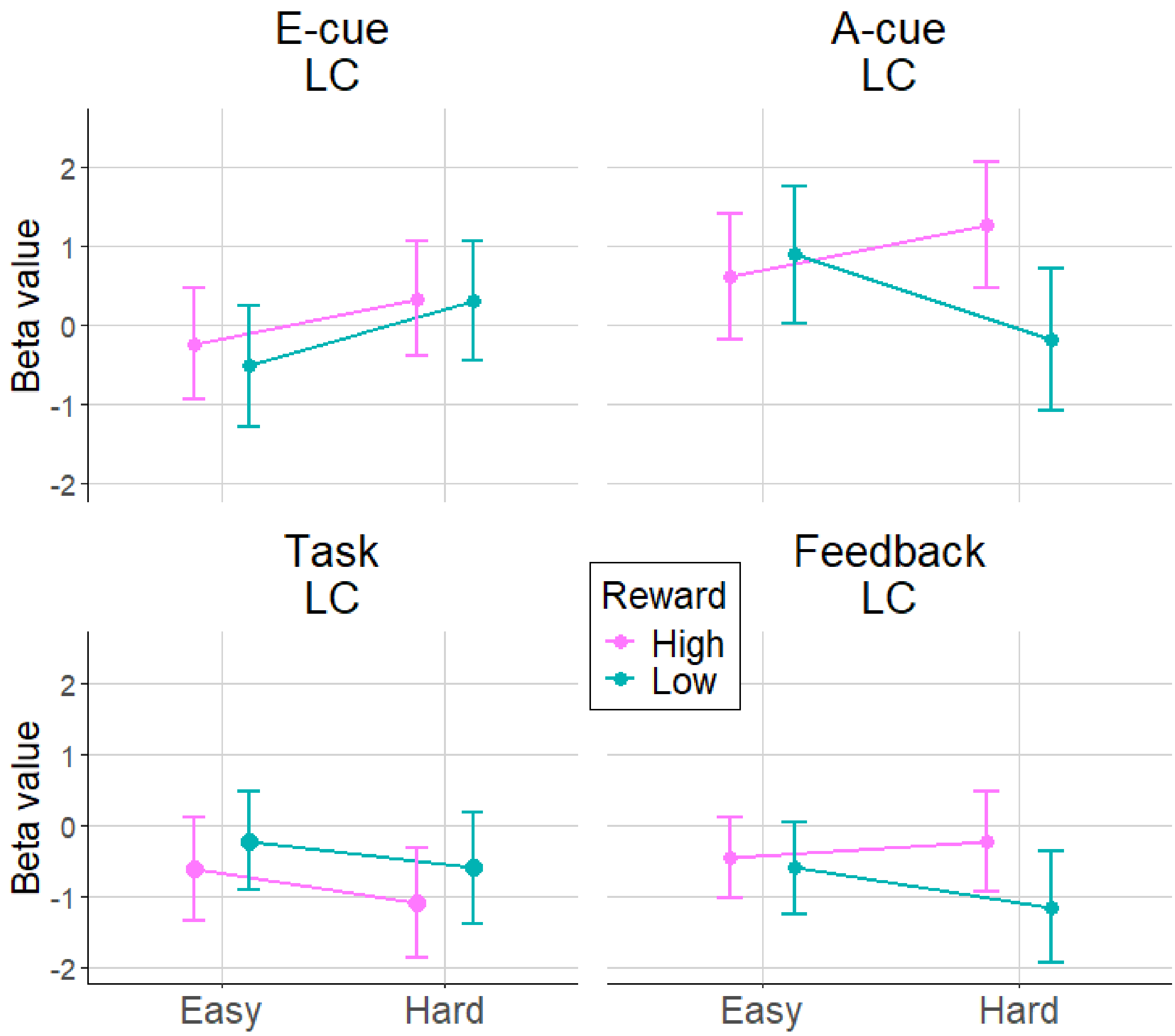

Note. Region-of-interest activity during evaluation cue (e-cue; top left), preparation cue (p-cue; top right), task (first target; bottom left), and feedback (bottom right). Error bars represent the estimated standard error of the mean. LC = locus coeruleus.

The effect of time

The fixed effect of *experiment half* was added to the ROI LMMs to tease out effects of time on the manipulation effects. The significant effects of both ACC and VTA disappeared with the introduction of *experiment half*. In the case of ACC, the interaction between reward and effort in the task phase was p = .053. Additionally, experiment half had a marginally

significant effect in the preparation and task phase, suggesting higher overall activity in the first half of the experiment in both cases. Similarly, VTA revealed a marginal effect of *experiment half* and its interaction with effort in the preparation phase.

In the evaluation phase, vmPFC revealed a significant interaction between effort and experiment half ($X^2[1] = 5.11$, $p < .05$): easy condition was associated with increased activity, but only in the second half ($b = 0.90$, SE = 0.32, $t = 2.80$, $p < .01$; first half: $b = -0.28$, SE = 0.32, $t = -0.86$, $p = .39$). In the preparation phase, a significant effect of experiment half ($X^2[1] = 4.71$, $p < .05$) suggested higher vmPFC activity in the first half of the experiment. VS conclusions were not affected by the introduction of experiment half in the evaluation phase. In the preparation phase VS exhibited an experiment half effect ($X^2[1] = 7.51$, $p < .01$), with first half being associated with more VS activity. An interaction between effort and experiment half ($X^2[1] = 5.00$, $p < .05$) suggested significantly higher activity in hard versus easy condition, only in the second half ($b = 0.80$, SE = 0.24, $t = 3.30$, $p < .01$; first half: $b = -0.05$, SE = 0.24, $t = -0.21$, $p = .84$). Task and feedback effects in both VS and VTA disappeared.

Effects within putamen and caudate

To investigate the manipulation effects within the wider control and reward networks, manipulation effects within the bi-lateral putamen (5-mm radius sphere ROIs, MNI center coordinates -19, 9, 2 and 17, 9, -2) and caudate (MNI center coordinates -10, 9, 2 and 10, 9, 2) were tested. In the evaluation phase caudate showed an interaction effect (left: $X^2[1] = 5.00$, $p < .05$; right: $X^2[1] = 5.35$, $p < .05$), responding more to high than low reward easy cues (left: $b = 0.61$, SE = 0.30, $t = 2.05$, $p < .05$) or showing non-significant but reverse simple effects, again preferred activation for high reward in easy condition, but for low

reward in hard condition (right). In the preparation phase, the left caudate showed no significant effects, while the right caudate revealed an interaction ($X^2[1] = 6.94$, $p < .01$), with preferred activity for high-reward, hard trial preparation (versus low-reward-hard: $b = 1.33$, SE = 0.32, $t = 4.08$, $p < .001$).

Both putamen responded preferentially during high-reward easy evaluation cues (left: $X^2[1] = 10.17$, $p < .01$; $b = 0.48$, SE = 0.24, $t = 2.01$, $p < .05$; right: $X^2[1] = 9.02$, $p < .01$; $b = 0.66$, SE = 0.22, $t = 2.99$, $p < .01$), and high-reward hard preparation (left: $X^2[1] = 9.45$, $p < .01$; $b = 0.94$, SE = 0.24, $t = 3.95$, $p < .001$; right: $X^2[1] = 7.20$, $p < .01$; $b = 0.70$, SE = 0.21, $t = 3.30$, $p < .01$).

Effects within a dACC salience network node

To separate our proactive effort-value integration effects of ACC from those related to more general salience tracking, the manipulation effects during evaluation and preparation were tested within a salience network region (MNI coordinates 6, 18, 30; 5-mm radius; Seeley et al., 2007). In the evaluation phase, this region showed an interaction between reward and demand ($X^2[1] = 5.26$, $p < .05$): the region responded preferentially to cues predicting a low- rather than high-reward, hard trial ($b = 0.47$, SE = 0.24, $t = 1.98$, $p < .05$). No effects were found in the preparation phase.
